# MagNet: Detecting Digital Presentation Attacks on Face Recognition

**DOI:** 10.3389/frai.2021.643424

**Published:** 2021-12-08

**Authors:** Akshay Agarwal, Richa Singh, Mayank Vatsa, Afzel Noore

**Affiliations:** ^1^ Indraprastha Institute of Information Technology Delhi, New Delhi, India; ^2^ Indian Institute of Technology Jodhpur, Jodhpur, India; ^3^ Texas A&M University, Kingsville, TX, United States

**Keywords:** digital threats, face recognition (FR), DeepFake detection, face swapping, face morphing attack

## Abstract

Presentation attacks on face recognition systems are classified into two categories: physical and digital. While much research has focused on physical attacks such as photo, replay, and mask attacks, digital attacks such as morphing have received limited attention. With the advancements in deep learning and computer vision algorithms, several easy-to-use applications are available where with few taps/clicks, an image can be easily and seamlessly altered. Moreover, generation of synthetic images or modifying images/videos (e.g. creating deepfakes) is relatively easy and highly effective due to the tremendous improvement in generative machine learning models. Many of these techniques can be used to attack the face recognition systems. To address this potential security risk, in this research, we present a novel algorithm for digital presentation attack detection, termed as MagNet, using a “Weighted Local Magnitude Pattern” (WLMP) feature descriptor. We also present a database, termed as *ID* ***Age*** *nder*, which consists of three different subsets of swapping/morphing and neural face transformation. In contrast to existing research, which utilizes sophisticated machine learning networks for attack generation, the databases in this research are prepared using social media platforms that are readily available to everyone with and without any malicious intent. Experiments on the proposed database, FaceForensic database, GAN generated images, and real-world images/videos show the stimulating performance of the proposed algorithm. Through the extensive experiments, it is observed that the proposed algorithm not only yields lower error rates, but also provides computational efficiency.

## 1 Introduction

The high performance of modern face recognition algorithms and the convenience of capturing face images have supported the applications to allow remote or unsupervised face images for authentication (Majumdar et al., 2017). For instance, now, online banking can be performed *via* face authentication. While this increases convenience and reduces fraudulent access, the security of these recognition systems is also an important task. Its importance can be observed from the launch of the Odin, IARPA project on biometric presentation attack detection^1^, which aims to protect the integrity of these systems.

Presentation Attacks are defined as “the attack on the system which in any way can affect the decision of a biometric system”. They can be broadly classified into two categories: digital and physical. Physical attacks include physical methods of deceiving the system, such as print and replay attack, 3D mask, and silicon mask attack. Digital presentation attacks include attacks such as morphing, swapping, and digital alterations. These attacks can be performed for multiple reasons, avoiding recognition, impersonating someone else’s identity, or multiple people sharing an identity. Researchers are recently studying adversarial attacks that are digital; however, they are targeted towards fooling specific deep learning architectures and are generally visually indistinguishable (Szegedy et al., 2013; Goodfellow I. J. et al., 2014; Goswami et al., 2018, 2019; Akhtar and Mian, 2018).

**FIGURE 1:**
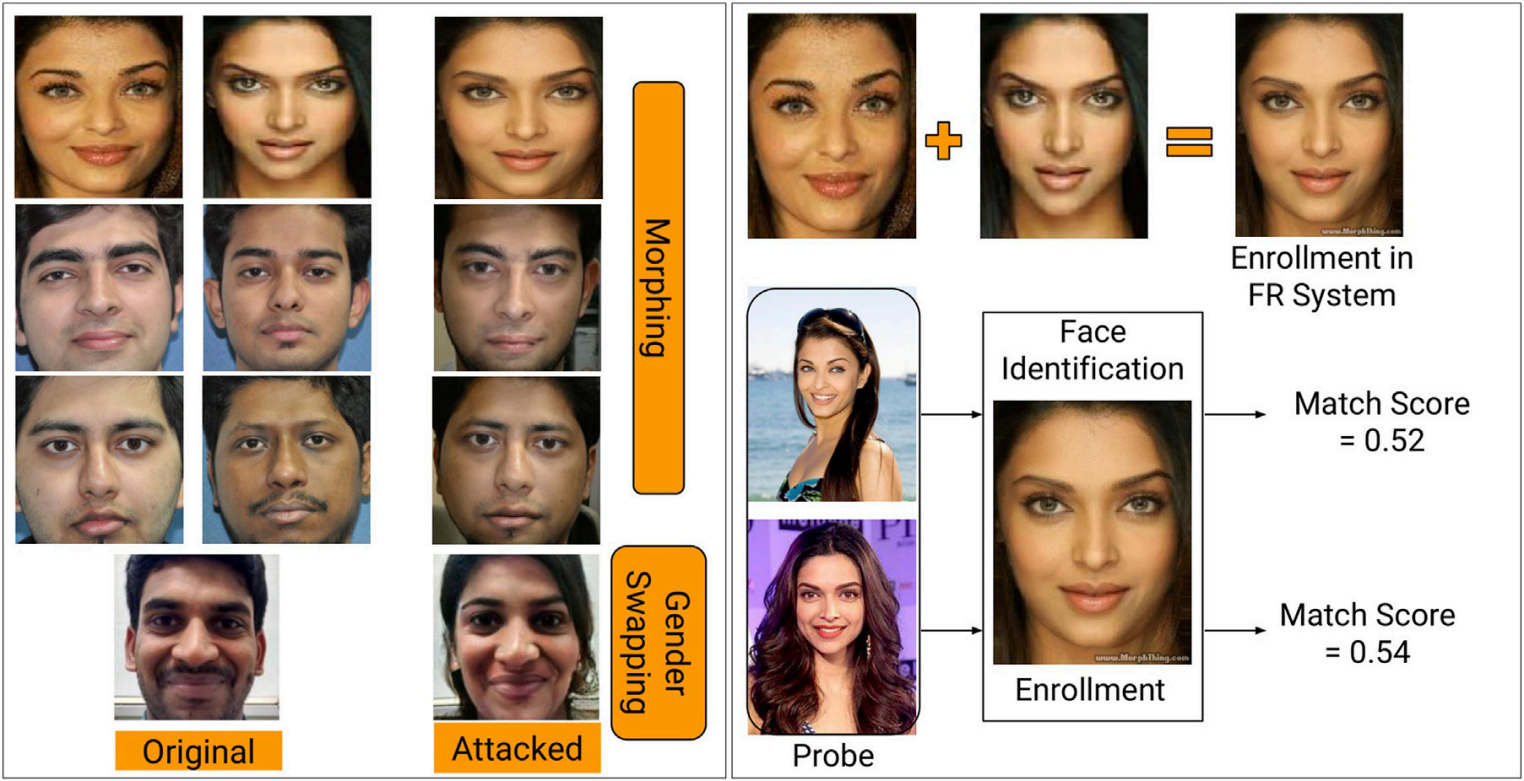
**(A)** Illustrating the effect of perceptible digital alterations on face images **(left)**. **(B)** Illustrates the effect when enrolled templates are modified *via* morphing. The bottom row shows that the two different identities can claim the same identity through enrolled morphed image. Experiments are performed using COTS Face Recognition (FR) **(right)**. Human identifiable images on the right side of the figure are taken from the Internet.

This paper focuses on detecting digital alterations in face images. The effect of face morphing in enrollment was first introduced by the International Organization for Standardization ISO 19792. In 2014, Ferrara et al. (2014) demonstrated the vulnerabilities of commercial face recognition systems towards morphed images. They also showed that these morphed images are challenging to be detected by face recognition experts and automatic algorithms (Ferrara et al., 2016). The popularity of face morphing applications worldwide can be observed by the fact that Facebook has acquired one of the famous morph applications called MSQRD. Figure 1 shows samples of digital alterations from multiple platforms. The first two columns show images of different subjects, and the third column represents the morphed image, which consists of similar facial features of both identities. The morphed image in the first row is generated using the Internet website called morphthing.com. The morphed images in the second and third rows are generated using the swap/morph feature of Snapchat application^2^. The gender swap image in the last row is generated using another popular mobile application called FaceApp^3^. Similarly, recently Instagram^4^ one of the most used social platforms for story and information sharing with more than one billion users, has launched face filters which can alter the facial properties in real-time.

The similarity of source and target images in Figure 1 (left) shows that digital attacks like morphing can be used to both elude and create a duplicate identity. To experimentally visualize the effect of morphing on face recognition, Figure 1 (right) shows the source and morphed images and the recognition outcome of a face recognition system. Using a commercial-off-the-shelf (COTS) recognition system, this example shows that the morphed image can successfully match its constituent source images. Inspired by the effectiveness of face morphing applications and the limitation of face recognition algorithms, this research focuses on designing a novel algorithm to differentiate between digitally attacked images and original/non-tampered images. As shown in Figure 1, different kinds of alterations introduce different effect on face images. The availability of limited databases in the literature makes it difficult to build a general digital manipulation detection algorithm. Moreover, the existing databases are captured using a limited number of subjects or neglected the significant social media platforms, which are easy to use by any novice user with or without malicious intent. Therefore, in this research, for the first time, we have utilized multiple social media platforms for the generation of altered face databases with various manipulation techniques such as swapping of faces and alteration of facial attributes such as age and gender. Further, to protect the integrity of automatic face recognition, we present a novel presentation attack detection algorithm that incorporates a new feature extraction algorithm. The contributions of this research can be summarized as follows:

• We propose a novel and computationally efficient feature descriptor, Weighted Local Magnitude Pattern (WLMP), which aims to encode the imperceptible artefacts that are embedded in the images after digital manipulation. It is our hypothesis that for detecting these manipulations, careful highlighting of the artefacts is necessary.
• We propose a novel MagNet algorithm for effectively differentiating between digital presentation attacks and original non-tampered videos/frames, using the proposed WLMP feature descriptor;
• We present a new database termed as “*ID* ***Age*** *nder*” - Digital Attack Face Database. It comprises of three different databases generated using three separate techniques: 1) face swap/morph feature of Snapchat, 2) Internet website *http://www.morphthing.com/* , and 3) gender and age swap/morph feature of face transformation application called FaceApp;
• The effectiveness of the proposed algorithm is demonstrated using a series of experiments, including comparison with state-of-the-art presentation attack detection algorithms and results on existing databases developed using state-of-the-art (SOTA) generative networks.

The structure of the paper is as follows: first, we present a comprehensive survey of existing algorithms developed for face morphing detection. Later, the proposed digital alteration detection algorithm is presented, followed by proposed database developed using multiple social media platforms. Along with the experimental protocols, we also showcase the impact of digital manipulation on face recognition through several experiments utilizing face recognition commercial software. Finally, results of manipulation detection on the proposed and existing databases are presented along with comparisons with existing state-of-the-art algorithms based on hand-crafted features and deep neural networks.

## 2 Related Work

Existing digital alteration generation techniques can be broadly divided based on the utilization of tools: 1) landmark-based sophisticated computer graphics algorithms and 2) deep generative networks. In this section, we first present an overview of the attack generation algorithms. Thereafter, the detection of these digitally altered images through existing techniques are discussed.

### 2.1 Generation

Ferrara et al. (2014) showed the effectiveness of morphing attack to gain illegal access to the system. Morphed images were generated using genuine face images of two different individuals. GNU image manipulation program V4.0 software was used for morphing. They selected the best-morphed images based on the match scores provided by the face recognition system. One major limitation of this research is the size of the database and the number of subjects used for evaluation. In 2016, Raghavendra et al. (2016) prepared a relatively large database of morphed images using a process similar to Ferrara et al. (2014). The database contains 450 morphed images generated by morphing two and three different face images. Instead of choosing the best-morphed image based on the score of the face recognition system, the mean output image is used as the best-morphed image. However, the database is not released to the research community.

Gomez-Barrero et al. (2017) showed that when a morphed image is used as the enrollment image in the recognition system, even with higher thresholds on the match score, more than one individual can share an identity. While the e-passport and e-Pass renewal in countries such as New Zealand use a digital copy of the face in its application process, several other countries still use the print of the face image and a scanned copy as part of their application process. Inspired by this process, Scherhag et al. (2017) prepared the morph attack database where first the morph images are printed using two different printers. Then two different kinds of scanners are used for digitizing the printed morphed images. The database consists of 693 morph images, out of which there are 231 digital attack images and 462 scan attack images. It was shown that 100% recognition is achieved when digital attack images match original images, while scan images yield more than 95% Impostor Attack Presentation Match Rate (IAPMR) using VeriLook SDK.

Robertson et al. (2017) performed a detailed study to demonstrate that face morphing can be a threat to face recognition systems. They performed three experiments; in the first two experiments, human examiners were asked to match two pairs of images. In the third experiment, smartphone face recognition is attacked by morphed images. The experiment showed that morph images that contain 50% features of both the genuine users could be matched with more than 68% acceptance rate. It implies that two different individuals can share the passport. In the third experiment, they performed face unlocking using three different images. With a 100% morphing level, one person’s identity is completely changed to a second identity, and a 91.8% acceptance rate was reported. With the 90% morph level, no significant drop in the acceptance rate is reported. Majumdar et al. (2019) have generated digitally altered faces by morphing the individual parts of faces and showed that not only are the commercial systems and humans vulnerable to morphing, but state-of-the-art CNN models are also demonstrated to be sensitive. Apart from utilizing the landmark-based computer graphics algorithms, recently, the generative networks (GANs) have been used for manipulating images. Rossler et al. (2019) have prepared the FaceForensics++ database using four state-of-the-art generative methods, namely, Face2Face, FaceSwap, DeepFakes, and Neural Textures. Li et al. (2020) have prepared the large-scale deepfake database to exhibit the characteristics in the images generally floating over the social networking sites. Korshunov and Marcel (2018) have prepared the DeepFake database using GAN. The database is prepared with two qualities of images: low and high. The vulnerability of VGG and FaceNet is shown with 85.62% and 95.00% false acceptance rates, respectively. Jiang et al. (2020) presented one of the largest face swapping database *via* a variational auto-encoder to enrich the quality of face swapped images.

While both landmark-based and generative network-based manipulation techniques effectively fool the face recognition algorithms, both these techniques come with certain limitations. The landmarks generation algorithm requires specialized knowledge and human intervention both for landmark selection and post-processing operations for effective blending; otherwise artifacts can be visually perceived. At the same time, generative networks are computationally expensive, require extensive resources, sensitive to geometric distortions and subject attributes such as gender and ethnicity. In this research, contrast to these existing algorithms, we have utilized the social media platforms, which neither require any specialization in their operation nor is computationally expensive. The proposed research also covers variety in terms of manipulation platforms and consists of variations in manipulation types.

### 2.2 Detection

The existing detection algorithms can be broadly divided into hand-crafted image features and deep learning-based algorithms. Scherhag et al. (2017) have performed morph attack detection using multiple-image descriptors, binarized statistical image features (BSIF), local phase quantization (LPQ), local binary pattern (LBP), and 2D fast Fourier transform (FFT), with support vector machine (SVM) classification. Neubert (2017) applied multiple image degradation on the images with the intuition that the morphed images suffer serious edge degradation to detect the attack. Similarly, for morph attack detection, Makrushin et al. (2018) have computed DCT coefficients from the JPEG compressed images and fit a logarithmic curve over the Benford features. Seibold et al. (2017) have used three pre-trained deep CNNs (AlexNet, GoogLeNet, and VGG19) to detect the morph attack on face recognition systems. The training of all three networks from scratch yields 4.4–7.4% higher false reject rate (FRR) than the pre-trained networks. Raghavendra et al. (2017b) performed feature fusion on the first fully connected layers of AlexNet and VGG19 to detect the morph attack. The proposed approach shows Bonafide Presentation Classification Error Rate (BPCER) value of 14.38%, 41.78%, and 28.76% at 5% Attack Presentation Classification Error Rate (APCER). Wandzik et al. (2017) shows the vulnerability of deep CNN based face recognition under morphing attacks. ResNet v1 shows 99.97% acceptance rate on original images; the acceptance rate drops down to 34.66% on morphed images blended with 0.5% probability of images. Raghavendra et al. (2017a) developed two versions (print and digital) of the database by taking the average of two face images and morphing them together. The detection of morph attack is presented using LBP features in YCbCr and HSV color space. Scherhag et al. (2018) have proposed reference and no-reference image-based morph detection using various texture descriptors such as LBP, BSIF, Speeded Up Robust Feature (SURF), and Histogram of Gradients (HoG). Nguyen et al. (2019a) proposed the multi-task network to detect manipulated face images while cropping out the manipulated region. In place of using a multi-task network, Dang et al. (2020) have used the attention mechanism to highlight the important region for forgery detection. Matern et al. (2019), and Yang et al. (2019) have argued that several visual features in the computer-generated images are found missing at teeth, eyes, head pose, and facial contours. These visual features can be effectively used for morphed image detection. Nguyen et al. (2019b) have used the capsule network on the extracted features of the VGG network for various fake face detection. He et al. (2019) have used multiple color spaces as input to CNN for useful feature extraction. de Lima et al. (2020) and Mas Montserrat et al. (2020) have used the combination of CNN and recurrent network to model the inconsistencies presented in the forged videos at the frame level. Liu et al. (2020) enhanced the global texture feature extraction capability of CNN through the insertion of Gram block. Li et al. (2018) have proposed a recurrent convolution network to detect the fake images generated using generative networks. They have analyzed the inconsistency in the eye region to detect whether the videos are real or fake. Akhtar et al. (2020) have performed the ablation study of various deep networks to detect manipulation in images.

Overall, existing works have demonstrated, beyond doubt, that “morphing” is a threat for face recognition systems. Several survey papers have also highlighted the vulnerability of face recognition algorithm against digital manipulation and limitations of existing detection algorithms (Akhtar et al., 2019; Scherhag et al., 2019; Singh et al., 2020; Tolosana et al., 2020; Venkatesh et al., 2020). The survey papers bring out the boundaries of existing detection algorithms such as non-generalizability against manipulation types and image resolution and computationally inefficiency. The results on the proposed database (refer Table 5) also establish the generalizability issue of existing image feature-based and deep networks-based detection algorithms against multiple manipulation types. Therefore, to advance digital attack detection, a computationally efficient classification algorithm is proposed and demonstrated to be efficient against various manipulation types in this research. The proposed digital presentation attack detection algorithm can surpass existing algorithms by a significant margin.

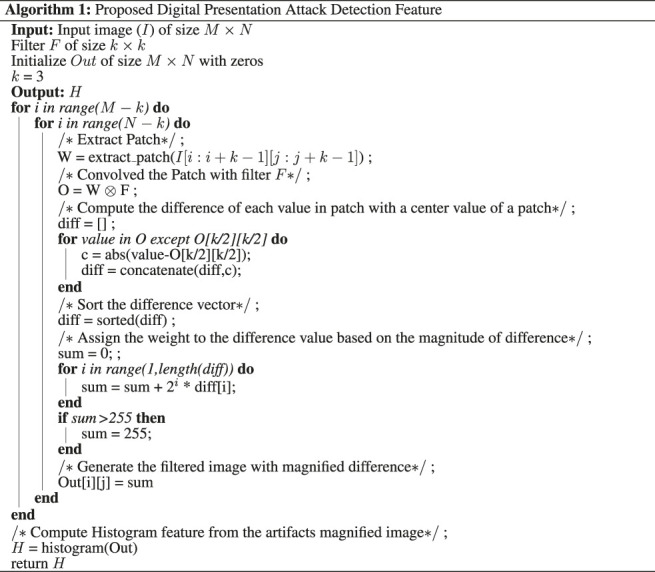

## 3 Proposed Digital Presentation Attack Detection Algorithm

**FIGURE 2:**
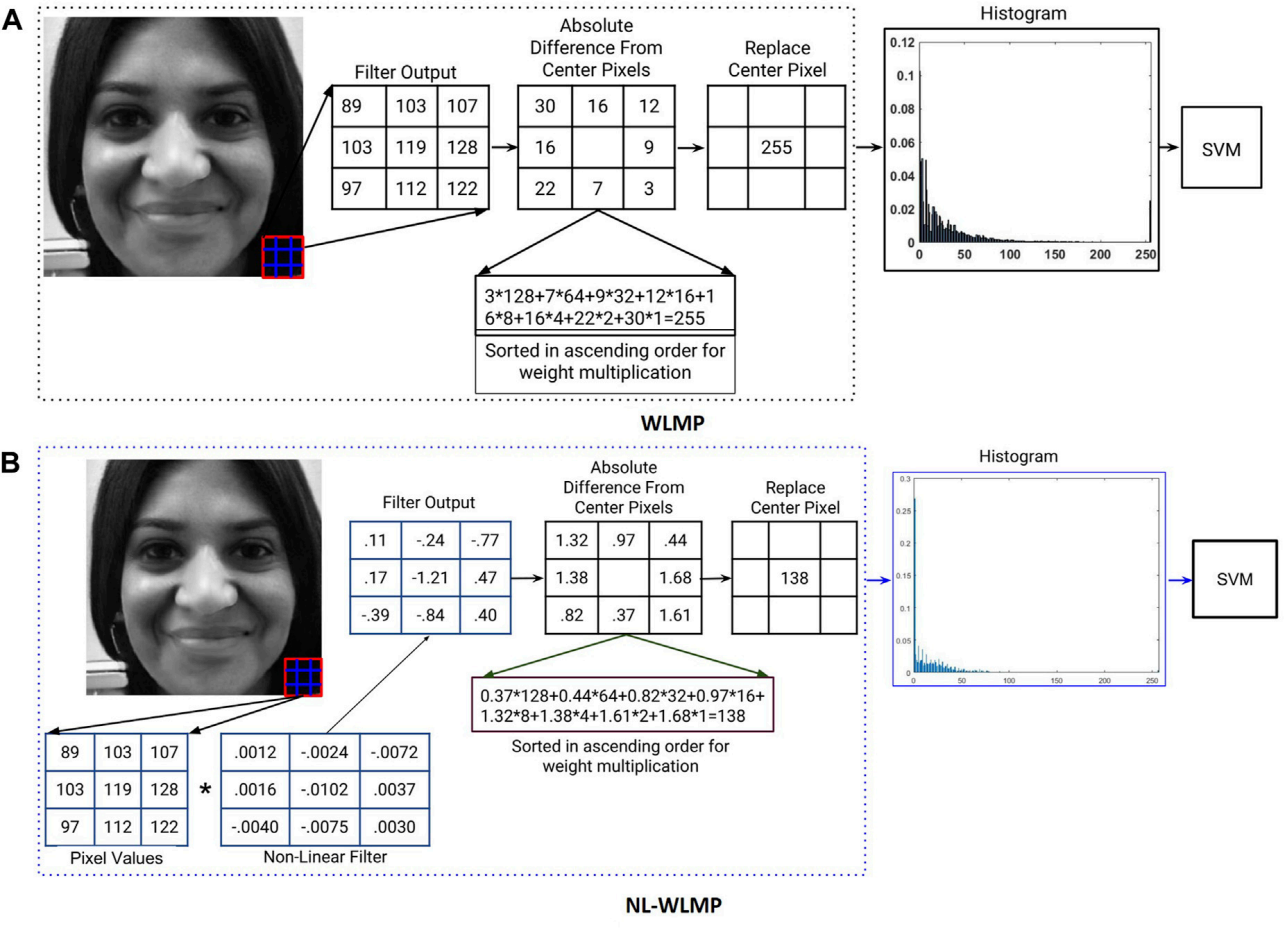
Illustrating the components involved in the proposed Weighted Local Magnitude Pattern (WLMP) and WLMP with convolution of image using non linearly learned filters (NL-WLMP) descriptor for digital attack detection.

We assert that digital alterations generally perform smoothing and blending to minimize the irregularities due to the differences in the source (and target) frames. It reduces the difference in neighboring pixel values in the resultant image. We hypothesize that it will be easy to detect digital alterations if we can highlight the irregularities between the neighborhood pixels and give weight according to the absolute values. Based on this hypothesis, we propose a novel feature encoding scheme for detecting digital alterations. As shown in Figure 2, the proposed algorithm is based on a novel feature encoding method termed as Weighted Local Magnitude Patterns (WLMP) for encoding digital alterations. The detailed description of the proposed WLMP and its variants is discussed below.

### 3.1 Weighted Local Magnitude Patterns

**FIGURE 3:**
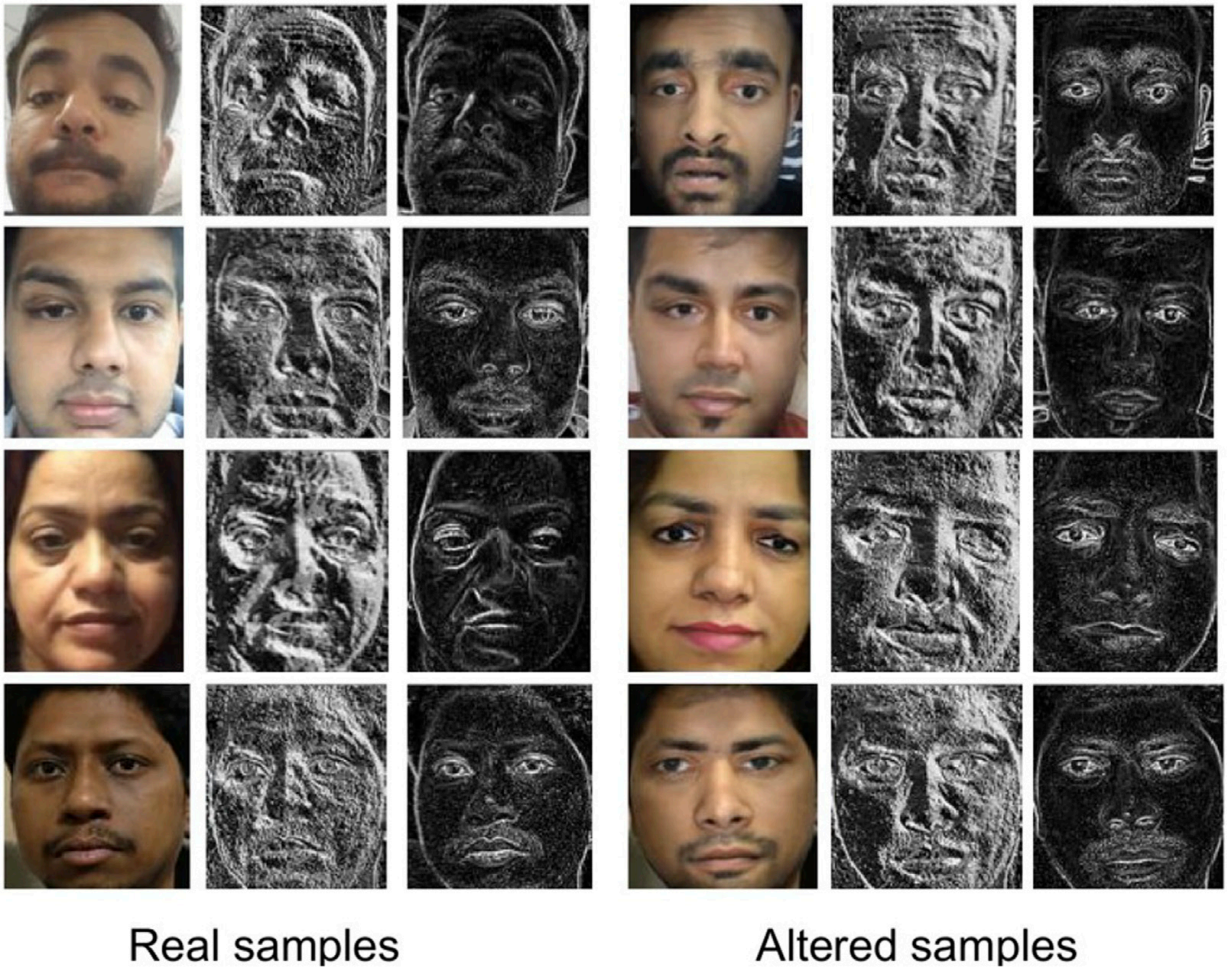
Illustrating the features obtained for real and altered samples from Snapchat database. In both real and altered samples, first column is the input images, second column is the LBP features, and last column is proposed feature images, respectively.

The input image is first tessellated into multiple patches of size 3 × 3. For each patch, the absolute difference between the center pixel and its neighborhood pixels is calculated. Since there are eight neighborhood pixels, there are eight difference values. The difference values are sorted in ascending order. Instead of binarizing the absolute differences, the sorted values are multiplied with 2^*p*^, where *p* = 0, …, 7 for eight neighborhood values. The motivation for sorting and multiplying is to give higher weight to the pixel, which has a value similar to the center pixel. The final output value is then mapped to a value in the range of 0–255 (i.e., any value greater than 255 is set to 255). Finally, a histogram feature vector is calculated based on the weighted local magnitude patterns of the image. The output images using the proposed feature descriptor are shown in Figure 3 along with the corresponding output obtained by LBP. It can be observed that the output images of the proposed feature retain the high-frequency information while reducing the low-frequency information. With images obtained from Snapchat’s swapped/morphed feature, facial keypoint regions such as eye, mouth, and nose are most affected, while the central region is well blended. It is clearly highlighted in the output images of the proposed feature extractor. Therefore, we postulate that for morphing related attacks, the proposed feature is better at detecting alterations than existing feature descriptors.

As shown in Figure 2A, we can visualize that the computation of WLMP descriptor includes convolving with an identity filter (i.e., convolving the patch with identity filter and then computing WLMP values). Convolution with linear/non-linear filters, as a pre-processing step, can help extract the features from the locally connected regions that can better differentiate original with altered images. For instance, morphing changes the local features of the face so that certain landmarks of the face exhibit the features of another person used for morphing. Further, while performing morphing operation, different facial structures undergo spatial changes to create an output image. A convolution operation with a filter before computing WLMP features can help highlight the subtle changes or artifacts in altered local regions. In the literature, it has been shown that filter based pre-processing/convolution helps in improved feature extraction (Randen and Husoy, 1999; Kang et al., 2017; Kuehlkamp et al., 2018). In this research, non-linearly learned filters obtained from deep learning model, GoogLeNet (Szegedy et al., 2015), are used for convolution/pre-processing.

### 3.2 WLMP with Non-linear Filter

Face morphing and digital alterations change the micro-texture property of the face region. Hence, the convolution of the input image with filters makes a strong case for encoding changes in the texture. For instance, while the software used for morphing blends two images nicely, some minute/micro-level artifacts can be observed around essential facial landmarks such as eye and mouth. Convolution with a learned filter can enhance these micro artifacts and help in computing representative WLMP descriptors.

The non-linear filters used in this research are obtained from GoogLeNet model (Szegedy et al., 2015). The filters at layer two, which are of size 3 × 3 are utilized from the pre-trained model^5^. The initial layer filter highlights the lower level features such as edges (Zeiler and Fergus, 2014), which might be one of the most reliable information in detecting digital alteration. Simultaneously, convolution with non-linear filters can help boost the detection of high-frequency information in the proposed WLMP descriptor. The pre-trained model provides four filters each with dimension 3 × 3 × 64, and we have taken the average around the third dimension to get the single filter of size 3 × 3. Therefore, four filters, i.e., average filter response from each of the four outputs of the model, are used in this research for convolution and feature extraction. Figure 2B shows the WLMP feature descriptor computation using a GoogLeNet filter and termed as Non-Linear Weighted Local Magnitude Pattern (NL-WLMP).

Algorithm 1 shows the pseudo-code of the proposed feature extraction algorithm for digital attack detection. In the case of WLMP, the filter of ones with size 3 × 3 is used; which is the patch of an image not convolved with any filter value. Whereas, in the case of NL-WLMP, CNN filters are first used to convolve an image patch. Each patch of an image is further used to magnify the subtle artifacts developed due to morphing through the weight assignment. A histogram feature vector is computed from the artifacts magnified image and used for digital attack detection.

### 3.3 MagNet: Proposed Algorithm for Digital Presentation Attack Detection

**FIGURE 4:**
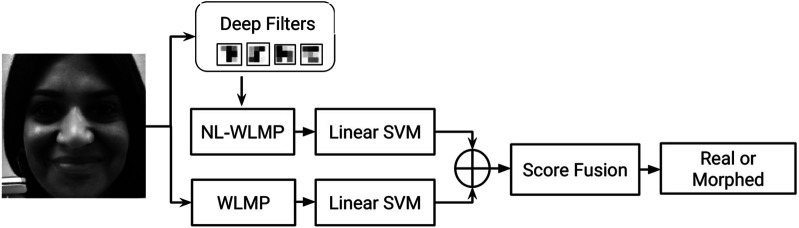
Proposed MagNet algorithm using fusion of WLMP and NL-WLMP.

WLMP and its variant, provides feature descriptor which can be fed into a 2-class (i.e., original vs attacked) classifier such as Support Vector Machine (SVM) (Cortes and Vapnik, 1995). Figure 4 illustrates the steps involved in the proposed digital presentation attack detection algorithm using WLMP. In the proposed algorithm, termed as MagNet, WMLP and NL-WLMP are individually used to compute the classification scores, which are then combined using score fusion. The score of each test frame is computed as the weighted score fusion of the scores computed from two different SVM classifiers, i.e. WLMP + SVM and NL-WMLP + SVM. Specifically,

• For each test image, WLMP feature descriptor is computed, and a score value is computed using the WLMP trained SVM classifier;
• Similarly, NL-WLMP feature descriptor is computed from the test image followed by the score which is obtained through NL-WLMP trained SVM classifier;
• Final score of a test image is computed using a weighted sum of the above two scores. The weights for fusion are computed over each database’s training/development set using a grid search. The fusion can be mathematically described as:

$$Final_{score}=w_{1}*\left[\begin{array}{c} y_{r1}\\ y_{p1} \end{array}\right]+w_{2}*\left[\begin{array}{c} y_{r2}\\ y_{p2} \end{array}\right]$$ (1)

where, *y*_*ri*_ and *y*_*pi*_ are the scores belonging to the real and presentation attack class computed using the *i*th classifier, respectively.

## 4 *ID* ***Agender!! Proposed Digital Attack Databases***

The second contribution of this research is *ID* ***Age*** *nder*, the proposed digital presentation attack database. *ID* ***Age*** *nder* ^6^ contains different subsets corresponding to different digital operations, and it is unique in terms of digital mediums, number of subjects, and type of alterations. There are several open-source algorithms and tools available for creating digitally altered images. However, Snapchat and morphthing.com are among the most popular and easily accessible tools for morphing or swapping face images. FaceApp, a mobile application, has recently become popular within a few months of its development for morphing gender and age characteristics. Since it is easy to navigate through these apps, non-technology savvy users can also efficiently use it to create various altered images. For example, a video where a woman swaps her face with Kardashians had been viewed more than 21,000 times in a week^7^. The face swap feature effectively changes the properties of the face, and by just looking at the altered face, it is difficult to determine whether it is real or altered. The proposed *ID* ***Age*** *nder* database^8^ consists of three different subsets: morphing, swapping, and FaceApp. The current literature ignores this aspect of readily available digital alteration on social media applications that neither requires sophisticated hardware nor knowledge to operate. The proposed research addresses this limitation and proposes an essential step towards securing face recognition systems from fake data of different kinds. The details of the three subsets are discussed in the following subsections.

### 4.1 Proposed Snapchat Face Swap Database

**FIGURE 5:**
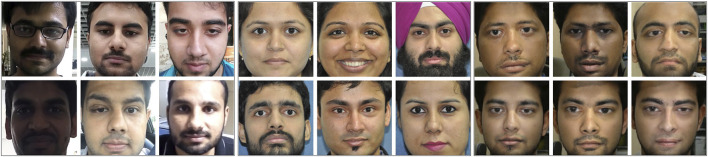
Sample images from the proposed Snapchat face swap database. **(A)** Sample bonafide images set **(left 3 columns)**, **(B)** sample images used for face swap **(middle 3 columns)**, and **(C)** morphed images from Snapchat **(right 3 columns)**.

The first subset of *ID* ***Age*** *nder* is a video database and consists of two parts: bonafide faces and morphed faces. Since morphing is applicable in both images and videos, and the Snapchat feature is more prevalent on mobile phones, the bonafide/genuine videos are captured using mobile phones. For every user, at least one video of around 6 s is captured using the front camera. In total, 129 bonafide videos are captured from 110 individuals over 2 months. These videos are captured in an unconstrained environment such as a natural outdoor, hallway, and inside office premises. Faces present in the videos are detected using the Viola-Jones face detector and normalized to 296 × 296 pixels. After face detection, the bonafide subset contains more than 30,000 face frames. Figure 5A shows sample images from the bonafide set captured in different illumination and background conditions.

To generate morphed faces, the face-swapping feature of Snapchat^9^, a popular social messaging app, is used. The steps involved in the process are as follows: first, the face is detected using the Viola-Jones face detector (Viola and Jones, 2004). To make the change more accurate and precise, key point location of the facial features such as eyes, mouth, and face boundary are detected using Active Shape Model (ASM) (Cootes et al., 1995). Once the facial keypoints are detected, a 3D mesh is generated, which fits the face properly and can move in real-time with changes in the face. The facial keypoints are detected from both the faces, and the central region is morphed from one image to the other. The boundary is then seamlessly blended to create the new morphed face image.

To prepare the morphed videos using Snapchat, two good quality frontal face images of 84 subjects (different from the bonafide faces) are captured in a semi-controlled environment. Samples of these images are shown in Figure 5B. These images are termed as the input gallery for face swapping/morphing. To create a morphed video, the Snapchat application requires the users to select the host video/image and an image they want to perform the face swap/morph. Using host videos from 31 subjects and input images from 84 subjects, 612 presentation (face swap) attack videos are prepared. Samples of morphed faces are shown in Figure 5C. Similar to bonafide faces, the detected morphed faces are normalized to size 296 × 296.

### 4.2 Proposed IdentityMorphing Face Swap Database

The second subset is prepared using the morphthing.com website by morphing several face images together. The morphing tool requires users to select the number of face images to be morphed together. It has the ability of morphing a maximum of four face images together. The face images in this database are captured in an unconstrained environment and have varying image quality. The real photos used in preparing the morph images are publicly available images of celebrities on this website.

**FIGURE 6:**
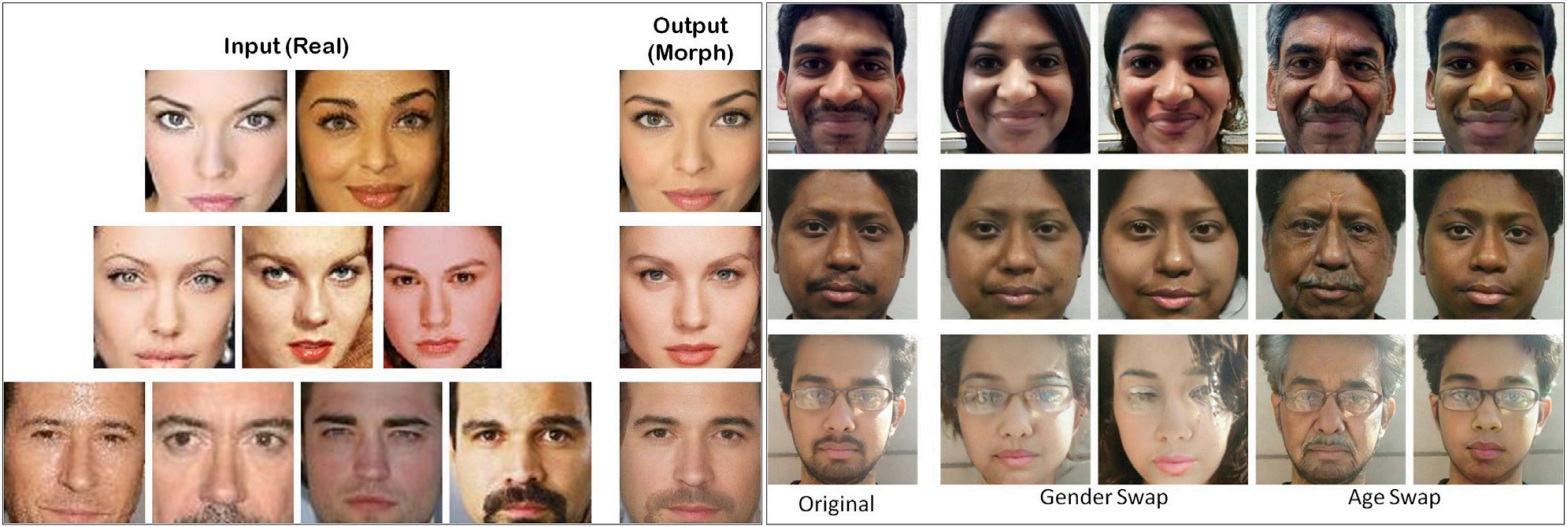
**(A)** Sample images from the proposed IdentityMorphing face swap database **(left)**. **(B)** Sample images showing age and gender swap using FaceApp **(right)**. Human identifiable images on the left side of the figure are taken from the Internet.

Table 1 shows comparing the proposed IdentityMorphing database with the existing morph databases. The proposed database has 1,200 morph images, out of which 500 images are generated by morphing two faces, 450 images by morphing three faces, and 250 images are generated through the morphing of four faces together. The proposed database is at least three times larger than existing databases in terms of the number of subjects and images. Sample images generated by morphing two to four faces together are shown in Figure 6. Figure 6A shows the morphed output images while Figure 6B shows the input real and output morph/swap images. The output morph faces have both visual and facial specific characteristics of all the faces used in its generation.

**TABLE 1:** Characteristics of the proposed and existing morph databases related to this research.

Database	Subjects	Faces morphed	Morphed images
Raghavendra et al. (2016)	110	2 and 3	450
Scherhag et al. (2017)	–	2	231
Proposed IdentityMorphing	545	2, 3, 4	1,200

### 4.3 Proposed FaceApp Database

The third subset of the proposed digitally morphed database is prepared using a mobile application, “FaceApp”^10^. FaceApp provides filters for gender morphing and age addition or subtraction. In this research, the database is prepared by morphing the gender of the person, adding the age to look older, and subtracting age to look younger.

To the best of our knowledge, this is the first work that presents a database having face images with altered age or gender. First, to create the digitally morphed images, good quality frontal face images of 125 subjects are captured in controlled illumination. To create the morph images, each user’s face image is given to FaceApp, and the image is morphed as per a given chosen filter. The filter represents the operation that will be performed on the input images; the process can be gender morphing and age addition/subtraction. The proposed database contains 375 digitally morphed face images.

## 5 Experimental Protocol and Performance Metrics

We also define a benchmark protocol that can be used to report and compare results on *ID* ***Age*** *nder*. In place of one particular train and test set, multiple fold cross-validation is performed to evaluate the performance of the algorithms.

### 5.1 Protocol for Snapchat Database

As explained earlier, the bonafide (real) subset of the Snapchat database contains 129 videos from 110 subjects, and the presentation attack subset contains a total of 612 videos from 31 subjects. Out of these videos, the real subset is divided into three random folds, where two folds contain 40 videos from 40 subjects each. The third fold contains 49 videos from 30 subjects. In the attack subset, the number of videos is large, and hence it is divided into 10 folds. Each fold of the attack subset contains 60 videos corresponding to three subjects except the last fold, which contains 72 videos from four subjects. Unlike three-fold cross-validation, where two folds are used for training and one for testing, one fold is used for training on this database, and the remaining folds are used for testing. The training set is reduced to evaluate the performance with limited training samples.

### 5.2 Protocol for IdentityMorphing Database

IdentityMorphing database contains 574 bonafide images and 1,200 morphed images. Out of these images, the real (bonafide) images are divided into three folds, and morphed images are divided into six random folds. Similar to the Snapchat database, one fold at a time is used for training while the remaining folds comprise the testing set.

### 5.3 Protocol for FaceApp Database

FaceApp database contains 250 bonafide images and 375 age and gender morph images. 375 morphed images are divided into three folds where each fold contains 125 images, and 250 bonafide images are divided into two folds with 125 images in each fold. Similar to the previous two databases, one fold is used for training, and the results are reported with remaining as the test set. The protocol of each of the proposed database is listed in Table 2. Real and attack subsets are divided so that equal samples (approximately) from both the classes can be used for training. For example, the IdentityMorphing database contains 545 real images divided into three folds, where each fold contains 180 images. Similarly, the attack set is divided into six folds, with each fold containing 180 samples (approximately). FaceApp database is divided into two real folds and three attack folds, where both types of folds include 125 samples of two classes.

**TABLE 2:** Experimental protocol of each of the proposed databases.

Database	Videos/Images	Real folds	Attack folds	Iterations	Metrics
Snapchat	129 Real and 612 Attack	3	10	30 (i.e. 3 × 10)	ACER and EER
Identity Morphing	545 Real and 1,200 Attack	3	6	18 (i.e. 3 × 6)	
FaceApp	250 Real and 375 Attack	2	3	6 (i.e. 2 × 3)	

### 5.4 Performance Metrics

Using the test set of the databases, the performance of presentation attack detection is reported in terms of the Equal Error Rate (EER) and Average Classification Error Rate (ACER). EER is defined as the point where the Bonafide Presentation Classification Error Rate (BPCER) is equal to the Attack Presentation Classification Error Rate (APCER). BPCER is the percentage of bonafide faces incorrectly classified as attack/altered faces, while APCER is the percentage of attack faces incorrectly classified as bonafide faces. To calculate the BPCER and APCER on the test set, a threshold value is selected based on the EER of the development set. In this research, half of the training set is used as the development set. ACER (%) is then computed as the average of BPCER and APCER.

$$ACER\left(\%\right)=\frac{BPCER+APCER}{2}*100$$ (2)

## 6 Effect of Swapping Attack on Face Recognition

To evaluate the effectiveness of the face swap feature as an attack on the face recognition system, we have performed two different experiments: 1) Various iOS devices are now equipped with the face unlock feature. Thus, the first experiment is face unlocking on iPhone/Android, and 2) face identification using a Commercial-Off-The-Shelf System (COTS), FaceVACS^11^. In the first experiment to unlock the iPhone/Android, a video of the morphed face prepared using an image of the genuine person enrolled in the mobile device is displayed in front of the mobile camera. It is interesting to observe that the face recognition algorithm in the iPhone cannot detect the attack and hence gets unlocked every time. It shows the vulnerability of face recognition in mobile devices towards digital attacks. In the second experiment, face identification is performed using a COTS system on all three databases, and the results are summarized in the next subsection.

### 6.1 Face Recognition with SnapChat and FaceApp Databases

**FIGURE 7:**
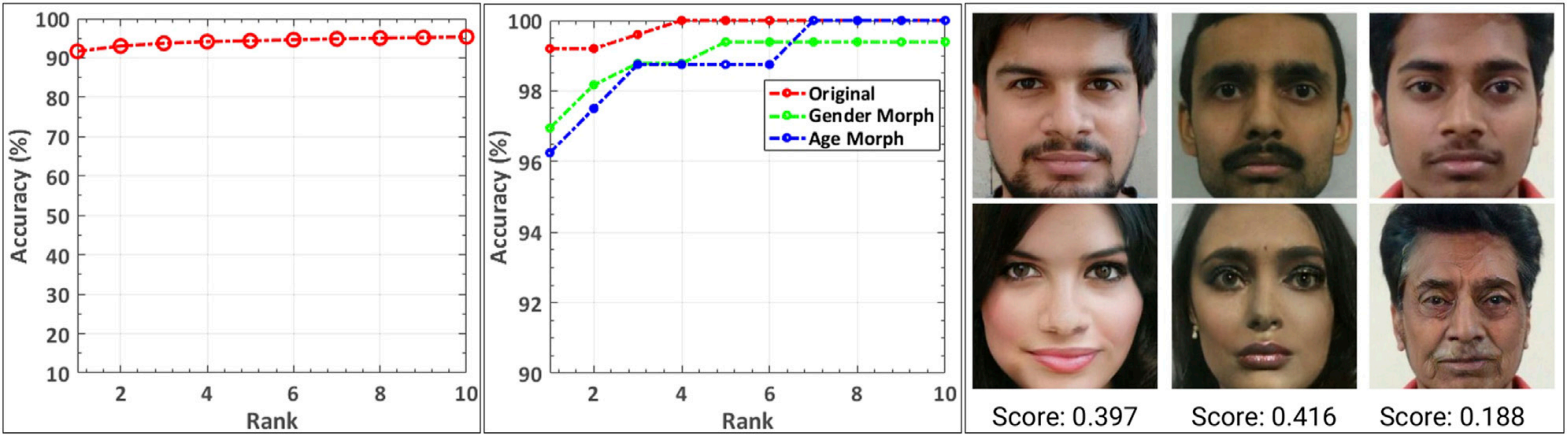
CMC plot for face identification. **(A)** Using Snapchat database **(left)**, **(B)** Using FaceApp database **(middle)**, and **(C)** Gallery and probe images with corresponding match scores obtained using COTS on the images from FaceApp database. The first row contains sample gallery images and the second row contains the corresponding age/gender morph probe image of that subject **(right)**.

To perform the Face Recognition (FR) experiment, another set of frontal images is collected from the individual whose images are used for creating the morphed videos. These images comprise the gallery for face identification. From each of the attack videos in the Snapchat database, 30 random frames are used as the probe set for the face identification experiment. Figure 7A shows the cumulative match characteristics (CMC) curve obtained for this experiment. It can be observed that 90% of the time, attack images are matched to enrolled gallery images at rank-1. Similarly, from the FaceApp database, one frontal image of each person, over which the gender and age morphing has been performed, is used as the gallery image. All the morphed images in the FaceApp database are used as the probe images. Figure 7B shows the effect of gender and age morph on face identification. When real images are matched to the gallery images, COTS shows more than 99% identification accuracy while morph images suffer a drop of 2–3% accuracy at rank-1. For the gender morph and age morph experiment, the digitally altered images corresponding to that particular subset are used as a probe. Figure 7 (right) shows the identification score of the probe images with respect to the gallery image of that subject. On matching real images without morphing, the highest score value of 0.999 is recorded, while with age and gender morphed images, the score reduces to 0.187 and 0.397, respectively. The low matching scores show the successful identity evasion using a digital attack.

### 6.2 Face Recognition on the IdentityMorphing Database

Like the Snapchat and FaceApp databases, the IdentityMorphing database contains the morph images of two, three, and four different identities that can be utilized for identity fraud. In this case, identity fraud can be described as the scenario where one probe image can be matched with different gallery images, or multiple individuals can share an identity in the gallery database. To perform this experiment, real photos of two individuals are used as gallery images, and morphed images generated using images of those individuals are used as the probe image. All the probe images shown in the figure yield a high match score value of 0.999 to both the gallery images.

## 7 Digital Presentation Attack Detection Results

The performance of the proposed algorithm is demonstrated on the *ID* ***Age*** *nder* digital attack face database. In the literature, face presentation attack detection (Galbally et al., 2014) using texture features has shown state-of-the-art performance (discussed in Section 2). Therefore, we have compared the performance of the proposed algorithm with the following state-of-the-art texture feature based algorithms along with CNN model.

• Local Binary Pattern (LBP) (Määttä et al., 2011)
• Rotation Invariant Uniform LBP (RIULBP) (Ojala et al., 2002),
• Complete LBP (CLBP) (Guo et al., 2010),
• Uniform LBP (ULBP),
• Local Phase Quantization (LPQ) (Ojansivu and Heikkilä, 2008),
• Binarized Statistical Image Features (BSIF) (Kannala and Rahtu, 2012), and
• Haralick + Redundant Discrete Wavelet Transform (RDWT) (Agarwal et al., 2016),
• Agarwal et al. (2017).
• Pre-trained VGG-16 Convolutional Neural Network (CNN) (Simonyan and Zisserman, 2015),
• Fine-tuned GoogLeNet CNN (Szegedy et al., 2015),
• XceptionNet (Rossler et al., 2019),
• ResNet-18 (Kumar et al., 2020),
• Sharp multiple instance learning (S-MIL) (Li X. et al., 2020).

CNN models are used in three settings: 1) ImageNet (Deng et al., 2009) pre-trained model is used as a feature extractor and binary SVM classifier is trained for digital morph attack detection, 2) CNN architectures used in recent studies such as ResNet-18 (Kumar et al., 2020), XceptionNet (Rossler et al., 2019), S-MIL (Li X. et al., 2020), and 3) ImageNet pre-trained models are fine-tuned using Adam optimizer for binary class classification. For fine-tuning batch size is set to 32 and initial learning rate of value 0.001 is used for training.

### 7.1 Results and Analysis on Snapchat Database

The protocol defined in Section 5 is used for experiments on the Snapchat swap database. Since the proposed database contains videos, the results can be measured in terms of video classification and frame classification. In the case of videos, the entire video is classified as bonafide or attack, whereas for frame-based, every frame is classified as bonafide or attack. The classification score of the video is calculated as the average of all the scores corresponding to frames of that video.

First, the performance of the proposed MagNet algorithm is evaluated on the Snapchat database and compared with state-of-the-art algorithms in literature (Scherhag et al., 2019). Figure 4 shows the proposed algorithm with the combination of micro-texture encoding using GoogLeNet filters and local magnitude pattern. On the Snapchat database, the fusion of WLMP and NL-WLMP yields the average EER of 13.2% and 18.0% for video and frame-based detection, respectively. However, the combination of WLMP and L-WLMP yields the EER of 14.3% and 21.0% for video and frame-based detection, respectively. Table 3 show the results of the proposed and existing features for digital presentation attack detection by face-swapping. The results are shown in Table 3, and the analysis is summarized below:

• The proposed MagNet algorithm which is a combination of WLMP and NL-WLMP shows an improvement of 34% in terms of EER from the second best-performing feature, i.e., LBP (i.e., hand-crafted filter-based algorithm) for video-based attack detection;
• The performance of L-WLMP in detecting digital attacks is similar without filtering (WLMP) and with linear filtering;
• The lower bit linear filters yield lower detection performance. One possible reason lies in the length of the feature vector. The feature vector of the *n* bit linear filter is 2^*n*^ *.* Hence, the higher bit filters have a large feature dimension;
• The non-linear filters which are learned using the deep CNN model, i.e., GoogLeNet, performs better than linear filters. The primary reason might be the richness of edge features preserved in the initial layers of the CNN model;
• We observe, from the experimental results, that the filtered WLMP versions yield better results than the original WLMP. Among the three variants, NL-WLMP outperforms WLMP and L-WLMP by at least 2.2% (in terms of EER). After score fusion of WLMP and NL-WLMP, the EER further reduces by over 2%;
• It is interesting to observe that the combination of Haralick + RDWT (Agarwal et al., 2016), which yields low EER on physical spoofing databases, provides the highest EER value of 25.6% in video-based digital attack detection. In the case of frame-based attack detection, the BSIF feature (linear filtering based algorithm) yields the lowest performance;
• The proposed WLMP feature histogram incorporates sorting in ascending order. However, when the difference values are sorted in descending order (i.e., higher weight to the least discriminant neighbor, the reverse of the WLMP), the EER increases from 18.2% to 25.5% and 24.5% to 29.3% for video and frame-based detection, respectively.
• We observe that the alterations performed *via* Snapchat are seamless, and therefore, it is a challenging task to differentiate between the bonafide and attack data.

**TABLE 3:** Performance (%) of the proposed and existing algorithm for video and frame based presentation attack detection on the proposed SnapChat database. The results are reported in terms of the average equal error rate and classification error rates along with standard deviation (±).

Input	Features	EER	ACER	APCER	BPCER
Video	LBP (Määttä et al., 2011)	21.7 ± 6.1	21.3 ± 5.9	16.51 ± 5.7	26.28 ± 6.1
	ULBP (Ojala et al., 2002)	24.5 ± 6.0	22.7 ± 5.8	12.17 ± 6.0	33.45 ± 5.9
	RIULBP Ojala et al. (2002)	24.7 ± 4.9	23.5 ± 4.7	16.12 ± 6.2	32.62 ± 3.2
	CLBP (Guo et al., 2010)	24.5 ± 6.1	24.8 ± 5.9	12.60 ± 5.2	37.16 ± 6.6
	Haralick + RDWT (Agarwal et al., 2016)	25.6 ± 7.2	24.5 ± 7.3	13.99 ± 8.5	35.24 ± 6.1
	BSIF (Kannala and Rahtu, 2012)	25.2 ± 9.1	24.9 ± 9.3	29.96 ± 13.0	20.07 ± 5.6
	LPQ Ojansivu and Heikkilä (2008)	22.9 ± 5.2	23.9 ± 5.0	33.33 ± 5.4	**14.58** ± **4.6**
	Agarwal et al. (2017)	18.2 ± 5.6	18.1 ± 5.5	6.71 ± 5.9	29.51 ± 5.1
	**Proposed (MagNet)**	**13.2** ± **3.4**	**12.9** ± **3.2**	**5.62** ± **2.8**	20.15 ± 3.6
Frame	LBP (Määttä et al., 2011)	27.1 ± 4.3	27.3 ± 4.1	21.83 ± 5.7	32.80 ± 2.5
	ULBP (Ojala et al., 2002)	29.0 ± 3.4	28.6 ± 3.3	17.68 ± 4.2	39.70 ± 2.4
	RIULBP (Ojala et al., 2002)	28.7 ± 3.7	28.7 ± 3.9	20.94 ± 5.1	37.06 ± 2.7
	CLBP (Guo et al., 2010)	28.7 ± 3.8	28.8 ± 3.6	18.88 ± 4.7	38.80 ± 2.5
	Haralick + RDWT (Agarwal et al., 2016)	28.9 ± 4.8	28.4 ± 4.6	21.82 ± 4.3	35.08 ± 4.9
	BSIF (Kannala and Rahtu, 2012)	30.2 ± 7.0	30.2 ± 6.9	31.45 ± 8.8	29.19 ± 5.0
	LPQ (Ojansivu and Heikkilä, 2008)	28.7 ± 4.0	30.4 ± 3.8	40.30 ± 3.6	**20.50** ± **4.0**
	Agarwal et al. (2017)	24.5 ± 5.1	25.4 ± 4.9	10.60 ± 5.9	40.26 ± 3.9
	**Proposed (MagNet)**	**18.0** ± **0.4**	**17.6** ± **0.3**	**8.72** ± **0.4**	26.47 ± 0.2

Result of the best performing algorithm is highlighted in bold.

Instead of using a handcrafted filter (or identity filter) and non-linear filters in the original WLMP (described in Section 3.1) and NL-WLMP respectively, a set of linear filters learned on patches of natural images are used. In this research, BSIF filters (Kannala and Rahtu, 2012) of size 3 × 3 are adopted for convolution. The reason for selecting the BSIF filters over other linear filters is the effectiveness in texture feature extraction. Linear filters used in this research are trained on 50,000 natural image patches (Hyvärinen et al., 2009). The learning of BSIF filters has two major steps: 1) whitening and dimensionality reduction using PCA (Wold et al., 1987), referred to as canonical preprocessing and 2) selection of independent statistical component of the filters by Independent Component Analysis (ICA) (Comon, 1994). The L-WLMP algorithm uses a different number of filters concerning bit sizes; therefore, the first experiment is performed to analyze the effect of bit length on the classification performance. Multi-bit BSIF magnitude patterns are extracted by concatenating the individual patterns obtained from each filter. The analysis in terms of multi-bit magnitude pattern is reported in Table 4.

**TABLE 4:** Classification performance of the proposed L-WLMP algorithm for video and frame based presentation attack detection on the proposed Snapchat database. The results are reported in terms of the equal error rate and average classification accuracy (%).

Input	BSIF filter bit	EER	ACER
Video	5	19.9	22.3
	6	21.8	23.2
	7	18.7	21.9
	8	18.2	20.6
	**7 and 8**	**17.6**	**19.5**
	5, 7, and 8	17.9	19.6
	5, 6, 7, and 8	18.0	20.0
	5, 6, 7, and 8 + PCA	23.2	24.5
Frame	5	26.2	27.3
	6	27.3	28.1
	7	25.7	27.2
	8	24.9	26.3
	**7 and 8**	**24.6**	**25.9**
	5, 7, and 8	24.7	26.0
	5, 6, 7, and 8	24.7	26.0
	5, 6, 7, and 8 + PCA	28.7	29.6

Result of the best performing algorithm is highlighted in bold.

We observe that higher multi-bit filters yield lower EER and ACER for both video and frame-based countermeasures. Feature fusion of two higher bit filters such as bits 7 and 8, yields the EER value of 17.6% and 24.6% for video and frame-based attack detection, respectively. Based on the performance of higher bit filters and combination experiments, we observed that fusion of features from bit 7 and 8 yields the best results. The detection performance of L-WLMP is 4–5% lower in comparison to the performance of NL-WLMP. In place of GoogLeNet filters, when we have used VGG-Face filters in NL-WLMP, the performance is 2–3% lower.

### 7.2 Results and Analysis on IdentityMorphing Database

The IdentityMorphing database experiments are performed based on the evaluation protocol provided in Section 5. The performance of the proposed algorithm is compared with LBP, BSIF, and LPQ, which were the top three features on the Snapchat database and most popular in the literature of digital attack detection. Table 5 shows the performance of the proposed and top three existing algorithms. The trend in performance is similar to the Snapchat database, and the observations are summarized below:

• The proposed algorithm yields an average EER value of 0.0% for frame-based attack detection. While the bonafide presentation classification error rate is 0.4%, which is the least among all the algorithms, the attack presentation classification error rate is 0.0%;
• The proposed features show an improvement of 78% in terms of ACER from the second best-performing feature, i.e., LBP;
• The ACER of the proposed algorithm is 0.2%, whereas the ACER of the CNN model is at least 9.7%;
• Microtexture features BSIF and LPQ, provide a high EER value of 6.2% and 6.1% on the IdentityMorphing database. BSIF shows the highest EER and ACER;
• Even at lower False Positive Rate (FPR), the proposed algorithm yields high True Positive Rate (TPR). The TPR of proposed, BSIF, and LPQ feature is 100%, 68%, and 1.1% receptively at 1% FPR;
• Poor performance of existing texture features and the CNN model shows the challenge in morph attack detection and importance of the proposed algorithm.

**TABLE 5:** Results of the proposed MagNet and existing algorithms on the proposed *ID* ***Age*** *nder* databases using frames/images as input.

Database	Features	EER (%)	ACER (%)
Snapchat	LBP Määttä et al. (2011)	27.1 ± 4.3	27.3 ± 4.1
	LPQ Ojansivu and Heikkilä (2008)	28.7 ± 4.0	30.4 ± 3.8
	BSIF Kannala and Rahtu (2012)	30.2 ± 7.0	30.2 ± 6.9
	VGG-16 Simonyan and Zisserman (2015)	17.7 ± 2.4	18.4 ± 2.3
	GoogLeNet Szegedy et al. (2015)	28.1 ± 5.1	29.1 ± 4.9
	S-MIL Li et al. (2020a)	**16.9** ± **3.6**	**18.2** ± **2.7**
	XceptionNet Rossler et al. (2019)	19.7 ± 4.7	23.6 ± 3.1
	ResNet-18 Kumar et al. (2020)	30.0 ± 5.9	31.6 ± 5.7
	**Proposed (MagNet)**	**18.0** ± **0.4**	**17.6** ± **0.3**
Identity Morphing	LBP Määttä et al. (2011)	**0.6** ± **0.2**	**0.9** ± **0.1**
	LPQ Ojansivu and Heikkilä (2008)	6.1 ± 0.3	6.2 ± 0.2
	BSIF Kannala and Rahtu (2012)	6.2 ± 0.4	6.2 ± 0.2
	VGG-16 Simonyan and Zisserman, (2015)	4.7 ± 1.1	9.7 ± 1.0
	GoogLeNet Szegedy et al. (2015)	12.3 ± 2.1	11.5 ± 0.9
	S-MIL Li et al. (2020a)	9.4 ± 1.2	11.7 ± 1.8
	XceptionNet Rossler et al. (2019)	7.9 ± 2.4	9.1 ± 1.1
	ResNet-18 Kumar et al. (2020)	8.5 ± 1.8	10.6 ± 1.2
	**Proposed (MagNet)**	**0.0** ± **0.0**	**0.2** ± **0.0**
FaceApp	LBP Määttä et al. (2011)	1.3 ± 0.8	2.7 ± 0.7
	LPQ Ojansivu and Heikkilä (2008)	**1.2** ± **0.4**	**1.3** ± **0.3**
	BSIF Kannala and Rahtu (2012)	30.3 ± 4.4	30.5 ± 4.5
	VGG-16 Simonyan and Zisserman (2015)	18.3 ± 2.5	21.4 ± 2.3
	GoogLeNet Szegedy et al. (2015)	23.7 ± 2.1	24.5 ± 2.7
	S-MIL Li et al. (2020a)	8.6 ± 1.8	12.3 ± 1.2
	XceptionNet Rossler et al. (2019)	10.2 ± 3.6	14.7 ± 1.8
	ResNet-18 He et al. (2016)	16.2 ± 3.3	14.8 ± 1.6
	**Proposed (MagNet)**	**0.4** ± **0.7**	**2.5** ± **0.4**

Result of the best performing algorithm is highlighted in bold.

Similar to the Snapchat database results, the results on Identity Morphing also show that the features that yield high performance on physical attacks might not necessarily be the best set of features for digital attacks.

### 7.3 Results and Analysis on FaceApp Database

The results on the FaceApp database using six fold cross validation as defined in Section 5 are given in Table 5.

• The proposed algorithm yields an average EER of 0.4% for frame/image-based attack detection. On the other hand, the BSIF histogram feature provides the highest EER and ACER values;
• The proposed algorithm shows the lowest APCER among all the features used for comparison, which is highly required in the high-security systems;
• The EER and ACER of the CNN model is at least 15.8% and 12.3% (absolute difference) higher than MagNet, respectively;
• TPR of the proposed algorithm, LPQ, and BSIF features at 1% FPR is 99.8%, 98.8%, and 2.7%, respectively.

### 7.4 Findings From Each Subset of Proposed Database

The results show that the detection of morphed images generated using the Snapchat image is challenging compared to other social media platforms, including morphthing.com and FaceApp. The prime reason for comparably lower detection performance might be attributed to the post smoothing performed by Snapchat to make the morphed images look ready for upload on the social media accounts. In contrast, morphthing.com does not bother about any post-processing by itself. The images on the website cover a broad spectrum of faces that differ in race and ethnicity; the swapping of faces of different groups left the artifacts higher than the same group’s morphing faces. On the other hand, FaceApp is a neural transfer-based app used for facial attribute conversions such as age and gender manipulation. That leads to the drastic change in the facial appearance and, hence, through the proposed algorithm, we aim to magnify these changes for better detection of manipulated images. The existing algorithms, including deep neural networks, do not aim to magnify such minute and facial attribute conversion artifacts and therefore perform significantly lower than the proposed “MagNet”.

### 7.5 Statistical Significance of Results

We next evaluate the statistical significance of results obtained using WLMP, L-WLMP, and NL-WLMP. McNemar test (McNemar, 1947) is performed on the test labels provided by the various descriptors mentioned above, with the null hypothesis being the results are statistically the same. The output of different descriptors can be reported in terms of the confusion matrix, which is then used to calculate the McNemar test statistics. The test statistics are given below:

$$H_{0}:P_{b}=P_{c},H_{1}:P_{b}\neq P_{c}$$ (3)

where, *H*_0_ is the null hypothesis, *P*_*b*_ and *P*_*c*_ are the probabilities of error yields by two different classifiers. The McNemar test is performed on four sets of descriptor pairs:

• WLMP and L-WLMP,
• WLMP and NL-WLMP,
• WLMP and Fusion of WLMP + L-WLMP, and
• WLMP and Fusion of WLMP + NL-WLMP

Using the McNemar test, we observe that the accuracies provided by these different descriptors are statistically significant by rejecting the null hypothesis at a 5% significance level.

### 7.6 Cross-Database Experiments

**FIGURE 8:**
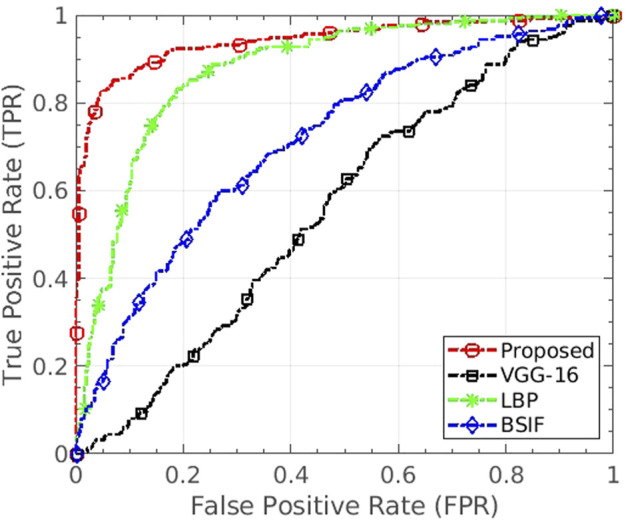
ROC of the cross-database experiment where the algorithms are trained on Snapchat and tested on FaceApp database. The proposed algorithm shows generalizability in handling unseen distortion type effectively.

We have also performed experiments under cross-database settings utilizing the proposed databases. When the proposed “MagNet” algorithm and VGG-16 model are trained on the Snapchat database and tested on FaceApp, the EERs of 12.0% and 46.1% are observed, respectively. The proposed algorithm also surpasses the CNN model in terms of area under the curve (AUC) as well by a significant value. It yields the AUC of 0.94, whereas VGG-16 CNN yields only 0.56. Compared to image features such as LBP, BSIF, and RDWT + Haralick (Agarwal et al., 2016), the AUC of the proposed algorithm is at least 7.0% better. Figure 8 shows the ROC curve of the experiment depicting the generalizability of the proposed algorithm in the handling of unseen manipulations. Similar higher performance has been observed when the proposed algorithm and CNN models such as VGG-16 and ResNet-18 are trained and tested on cross-database settings, including Snapchat vs IdentityMorphing and IdentityMorphing vs FaceApp.

Besides utilizing the proposed databases for cross-database evaluation, we have examined the robustness of the proposed detection algorithm on the existing database. The DeepFake (Korshunov and Marcel, 2018) database contains the face morphed images generated using generative adversarial networks (GANs). In total, the database contains 640 tampered videos in low and high quality. When the proposed algorithm trained on the Snapchat database is tested on the morphed images of Deepfake, presentation attack detection error (APCER) of value 0.0% is achieved. In other words, on both quality subsets, the proposed algorithm yields perfect detection performance. The performance of the proposed algorithm is 47.80% and 28.2% better than the deep neural network namely VGG-Face (Parkhi et al., 2015) and recently proposed PFTD algorithm (Majumdar et al., 2019).

#### 7.6.1 Computational Efficiency

Finally, computationally, the proposed algorithm requires 0.2 s to process an input image on a workstation with i7 processor and 16 GB RAM (without any GPU and parallel processing), whereas CNN based approach requires 3.0 s (using the same computing platform).

## 8 Real World Evaluation

We have also evaluated the performance of the proposed detection algorithm on artificially generated fake videos. The state-of-the-art image and video generation technologies can be efficiently used to create artificial images and videos (Goodfellow et al., 2014). The popularity and easy availability of these tools, such as DeepFakes and Fake APP^12^, have led to dramatic increase in fake videos on the Internet. By pursuing the misuse or the dark side of these practical algorithms, people have created fake porn videos of not only famous celebrities^13^ but it is also being used for harassment of women^14^. To evaluate the performance of the proposed algorithm on this attack, we collected real and swapped faces from online YouTube videos^15^. In total, 2,338 real and deepfakes with swap faces are collected.

Protocol and Result: 2,388 faces of each class are divided into five random folds, where each time four folds are used for training the classifier, and the images from the remaining one fold are used for testing. The average EER value with standard deviation is calculated to report the performance of the proposed algorithm. The proposed algorithm yields 2.05 ± 0.49% EER on this new online collected database. While most of the samples are correctly classified, further analysis of misclassified samples shows that low image quality is a covariate in attack detection. If the image is of low quality, it becomes challenging to determine whether the image is attacked or not. In comparison, the second-best EER of 6.89 ± 0.10% is achieved using CNN based digital presentation attack detection.

### 8.1 Experiments on Existing Database

In the previous sections, the experiments are conducted on either proposed databases collected in our lab or on the videos collected from YouTube. However, the tremendous improvement of the machine learning algorithms such as generative adversarial networks (GANs) has made the generation of synthetic images, transferring attributes among faces, or morphing two faces together an easy task. By utilizing these algorithms, several challenging digitally tampered face databases are prepared in literature. Therefore, to further show the strength of the proposed face morphed detection algorithm, one of the challenging large-scale morphed databases, namely FaceForensics (Rössler et al., 2018) is also used along with the database prepared by Jain et al. (2018). The FaceForensics database (Rössler et al., 2018) contains half a million edited face images. This database contains 704 and 150 training videos of real and morphed classes each. For evaluation, subject independent real and morphed videos of 150 subjects are used. For the collection of morphed videos Face2Face reenactment technique (Thies et al., 2016) is used.

The morphed image detection results of MagNet using the pre-defined database protocol and existing algorithms range from handcrafted features to deep CNN features reported in Table 6. In Steganalysis + SVM, the co-occurrence features from horizontal and vertical edge images are captured. This technique won the first challenge of image forgery detection (Cozzolino et al., 2014). Cozzolino et al. (2017) extracted handcrafted features from a deep learning architecture to detect morphed images. Bayar and Stamm (2016) have proposed 18 layers of convolutional neural network for face morphing detection. The network consists of a constrained convolutional layer that is designed to suppress the high-level information. Rahmouni et al. (2017) have extracted four statistical features from the CNN architecture. The features of the first fully connected layer of VGG-19 and AlexNet are concatenated by Raghavendra et al. (2017b). The concatenated elements are then passed to the Probabilistic Collaborative Representation classifier for real and morphed image classification. Zhou et al. (2017) have performed fusion of the scores obtained from two CNNs: GoogLeNet Inception V3 and triplet CNN. The state-of-the-art Xception network is fine-tuned for face morphing detection in a transfer learning fashion. The results of existing algorithms are taken from Rössler et al. (2018). The proposed algorithm either outperforms existing algorithms, including CNN features, statistics, and steganalysis features or performs comparable to them. For instance, the algorithm by Raghavendra et al. (2017b), which fuses the features from two CNNs, i.e., AlexNet and VGG-19, has 2.3% lower detection accuracy than the proposed MagNet. Further, on frame based evaluation: where every single frame is classified as real or morphed, the proposed MagNet algorithm yields 1.00%, 1.07%, and 0.93% ACER, BPCER, and APCER respectively. The MagNet algorithm shows the EER value of 1.67% and 0.00% for the frame and video-based evaluation on the FaceForensics database, respectively.

**TABLE 6:** Classification accuracy of the proposed and existing algorithm for video based morphing attack detection on the FaceForensics database.

Algorithm/Network	Accuracy %
Steganalysis Features + SVM Fridrich and Kodovsky, (2012)	99.40
Cozzolino et al. (2017)	99.60
Bayar and Stamm (2016)	99.53
Rahmouni et al. (2017)	98.60
Raghavendra et al. (2017b)	97.70
Zhou et al. (2017)	99.93
XceptionNet Chollet (2017)	99.93
MesoNet Afchar et al. (2018)	96.80
VGG-16 Simonyan and Zisserman (2015)	99.50
ResNet-50 He et al. (2016)	99.93
ResNet-152 He et al. (2016)	99.89
Multi-patch ResNet-18 Kumar et al. (2020)	99.96
**Proposed (MagNet)**	**100.00**

Result of the best performing algorithm is highlighted in bold.

### 8.2 Experiments with GAN Generated Images

Jain et al. (2018) have performed attribute transfer using StarGAN (Choi et al., 2018) and generated 18,000 face images. Nine different attributes, such as hair, age, and gender, are transferred. For the classification of GAN vs real images, a similar protocol mentioned by the authors is followed. The database is divided into training, validation, and testing set. The testing set contains random 1,500 real and 1,000 GAN generated images, while the validation set contains 500 images of both classes (i.e., real and GAN). The detection performance of the proposed and existing algorithms is summarized in Table 7. The proposed algorithm outperforms the existing state-of-the-art algorithms by at least 0.3%. Other than the detection accuracy, the EER, APCER, and BPCER of the proposed algorithm are 0.0%, 0.0%, and 0.4%, respectively.

**TABLE 7:** Classification accuracy of the proposed and existing algorithm for GAN generated images.

Algorithm	Accuracy %
Bharati et al. (2016) (Unsupervised DBM)	81.90
Bharati et al. (2016) (Supervised DBM)	87.10
Jain et al. (2018) (Thresholding)	99.48
Jain et al. (2018) (SVM)	99.65
**Proposed (MagNet)**	**99.96**

Result of the best performing algorithm is highlighted in bold.

The proposed algorithm is also evaluated on the images prepared by Jain et al. (2020) using super-resolution GAN (Ledig et al., 2017). Similar to StarGAN, nine facial attributes are transferred on the CelebA database (Liu et al., 2018). The detection performance is measured using a similar protocol used for starGAN images. The database is divided into training (27,500 images), validation (1,000 images), and testing images (2,500 images), respectively. The proposed algorithm yields 99.88% detection accuracy with 0.08% EER, 0.06% BPCER, and 0.20% APCER values.

### 8.3 Robustness to Unseen GAN Models

We have also evaluated the generalizability of the proposed MagNet algorithm using images generated from unseen GAN models. In this setting, the images generated using one type of GAN are used for training, while testing is done on different GAN images. When StarGAN images are used for training, and SRGAN images are used for testing, the MagNet yields 95.32% detection accuracy with 3.52% EER, 2.27% BPCER, 6.90% APCER, and 4.58% ACER values.

## 9 Conclusion

A rich literature on physical presentation attack detection shows the maturity of algorithms in protecting the face recognition systems. However, these systems are still vulnerable to digital attacks such as morphing. As a first contribution, this paper extends the research on digital attacks and presents the *ID* ***Age*** *nder* database of morphed faces using three sources of alterations, Snapchat, FaceApp, and MorphThing.com. Next, to protect the integrity of face recognition algorithms, a new computationally efficient digital presentation attack detection algorithm is proposed using a novel descriptor, termed as Weighted Local Magnitude Patterns. The proposed algorithm achieves lower error rates compared to several existing approaches on the proposed databases. The strength of the proposed algorithm is also demonstrated on the face-swap images created using generative adversarial networks. The generalizability of the proposed algorithm in handling unseen databases, attack types, and generative networks shows its merit for real world deployment. We believe the performance of the proposed algorithm can be improved under cross-database and cross attack settings. Further, the NL-WLMP can utilize a learning algorithm to obtain the specific filters that highlight the attack or database-specific features. Finally, the approach can be easily extended to other biometric modalities such as iris (Kohli et al., 2016).

## Data Availability

The raw data supporting the conclusion of this article will be made available by the authors, without undue reservation, for academic research.

## Footnotes

1
https://www.iarpa.gov/index.php/research-programs/odin.

2
https://store.snapchat.com/.

3
https://faceapp.com/.

4
https://www.instagram.com/?hl=en.

5
http://www.vlfeat.org/matconvnet/pretrained/.

6 This database will be made available to the research community *via* http://iab-rubric.org/resources.html.

7
https://tinyurl.com/k6nfly9.

8 Written informed consent was obtained from the individual(s) to publish any potentially identifiable images or data included in this article.

9
https://www.snapchat.com/.

10
https://play.google.com/store/apps/details?id=io.faceapp&hl=en.

11
http://www.cognitec.com.

12
https://www.fakeapp.org/.

13
https://tinyurl.com/y9ydm4yy/.

14
https://indianlawwatch.com/latestnewsupdates/bombay-high-court-asks-government-to-act-against-bot-that-turns-womens-photos-into-nudes/.

15
https://www.youtube.com/watch?v=-QvIX3cY4lc.
